# Assessment of the accuracy and stability of frameless gamma knife radiosurgery

**DOI:** 10.1002/acm2.12365

**Published:** 2018-06-03

**Authors:** Hyun‐Tai Chung, Woo‐Yoon Park, Tae Hoon Kim, Yong Kyun Kim, Kook Jin Chun

**Affiliations:** ^1^ Department of Neurosurgery Seoul National University College of Medicine Seoul Korea; ^2^ Department of Radiation Oncology Chungbuk National University College of Medicine Cheongju Korea; ^3^ Department of Nuclear Engineering Hanyang University College of Engineering Seoul Korea; ^4^ Department of Accelerator Science Korea University Sejong Campus Sejong‐ro, Sejong Korea

**Keywords:** frameless radiosurgery, gamma knife, image coregistration, positional accuracy, whole procedure test

## Abstract

The aim of this study was to assess the accuracy and stability of frameless gamma knife radiosurgery (GKRS). The accuracies of the radiation isocenter and patient couch movement were evaluated by film dosimetry with a half‐year cycle. Radiation isocenter assessment with a diode detector and cone‐beam computed tomography (CBCT) image accuracy tests were performed daily with a vendor‐provided tool for one and a half years after installation. CBCT image quality was examined twice a month with a phantom. The accuracy of image coregistration using CBCT images was studied using magnetic resonance (MR) and computed tomography (CT) images of another phantom. The overall positional accuracy was measured in whole procedure tests using film dosimetry with an anthropomorphic phantom. The positional errors of the radiation isocenter at the center and at an extreme position were both less than 0.1 mm. The three‐dimensional deviation of the CBCT coordinate system was stable for one and a half years (mean 0.04 ± 0.02 mm). Image coregistration revealed a difference of 0.2 ± 0.1 mm between CT and CBCT images and a deviation of 0.4 ± 0.2 mm between MR and CBCT images. The whole procedure test of the positional accuracy of the mask‐based irradiation revealed an accuracy of 0.5 ± 0.6 mm. The radiation isocenter accuracy, patient couch movement accuracy, and Gamma Knife Icon CBCT accuracy were all approximately 0.1 mm and were stable for one and a half years. The coordinate system assigned to MR images through coregistration was more accurate than the system defined by fiducial markers. Possible patient motion during irradiation should be considered when evaluating the overall accuracy of frameless GKRS.

## 1 INTRODUCTION

**Figure 1:**
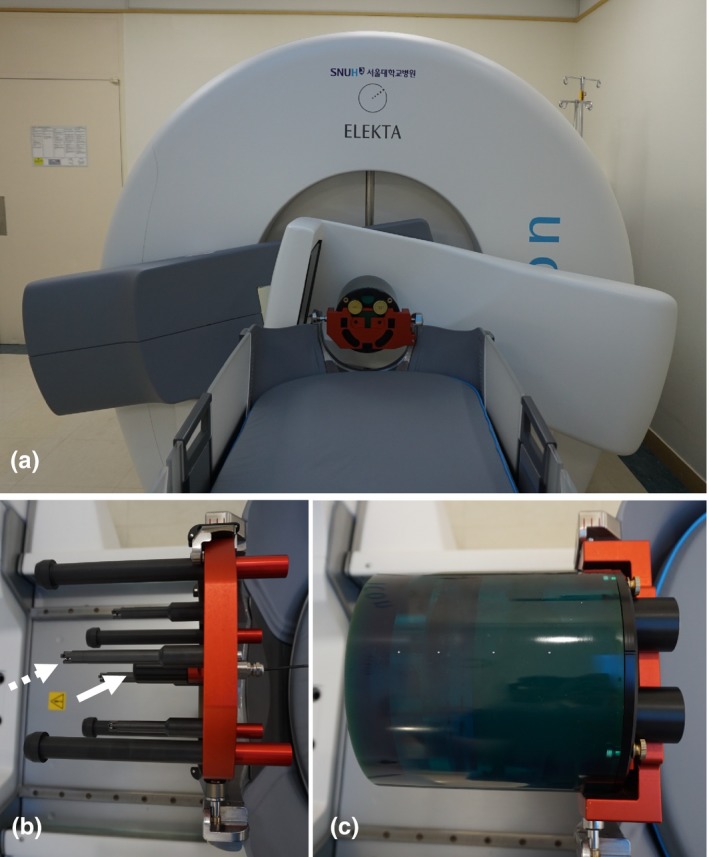
(a) Gamma Knife Icon^TM^ and its CBCT system in an imaging setup. (b) A vendor‐provided tool was used to measure deviations of the radiation isocenter and the accuracy of CBCT images. The white arrow indicates the diode detector used to measure dose distributions, and the dotted arrow indicates one of the four ball bearings employed to assess the geometrical accuracy of the CBCT images. (c) A CatPhan^®^ 503 phantom was used for CBCT image quality assessment.

Frameless gamma knife radiosurgery (GKRS) can be performed with the latest gamma knife model, Gamma Knife (GK) Icon^TM^ [Fig. 1(a)]. A stereotactic coordinate system necessary for frameless GKRS is assigned by coregistration of clinical magnetic resonance (MR) and/or computed tomography (CT) images with cone‐beam computed tomography (CBCT) images obtained with a CBCT system. After a treatment plan is completed based on this coordinate system, the patient is fixed by a mask, and another set of CBCT images is obtained such that the deviations in patient position can be automatically corrected. During irradiation, the motion of the patient is monitored by a high‐definition motion management (HDMM) system. When the motion is greater than a predefined limit for more than 2 s, the sources are retracted to their temporary storage positions. If the motion is maintained at levels greater than the limit for 30 seconds, the patient couch moves out, and CBCT imaging is repeated to correct for the movement. The overall accuracy of the frameless GKRS depends on numerous factors that can be classified into three groups: radiation‐related factors, image‐related factors, and patient motion. Given that the accuracy of frame‐based GKRS, such as the absolute dose measurement, treatment planning program accuracy, frame accuracy, and MR image distortion, has been studied in many previous works;^1^, ^2^, ^3^, ^4^, ^5^, ^6^, ^7^, ^8^, ^9^, ^10^, ^11^, ^12^ this work focused primarily on the accuracy aspects unique to frameless GKRS. The accuracy and stability of the radiation isocenter, the accuracy of patient couch movement, the accuracy and stability of CBCT images, the accuracy of image coregistration, and the accuracy of the HDMM system were assessed. whole procedure test of the geometrical accuracy of frameless irradiation with CT images were also performed. The stability of the measured values over one and a half years after installation was also analyzed. During the same period, independent periodic management was performed by the manufacturer at 6‐month intervals. The assessment of patient motion during irradiation was excluded in this work because such motion is patient‐specific and cannot be evaluated in phantom studies. The results were compared with the accuracy and image quality obtained at the time of machine commissioning^13^ and with the short‐term stability of the CBCT system.^14^

## 2 MATERIALS AND METHODS

### 2.A Accuracy of the radiation isocenter and patient couch movement

The radiation isocenter of a gamma knife should coincide with the center of the patient‐positioning system (PPS). The deviation between the radiation isocenter and the center of the PPS was measured using radiochromic films (GafChromic^TM^ EBT3; Ashland Specialty Ingredients, NJ, USA) following a vendor‐recommended procedure described by Novotny et al.^15^ In brief, a film was set within a special tool provided by the manufacturer, and the center of the PPS was marked by a sharp pin. Then, the film was irradiated using the 4‐mm collimator of a gamma knife and scanned. The deviation of the radiation isocenter, defined as the difference between the center of the radiation peak and the PPS center marked on the film, was measured. Three films were used in each test, and the procedure was performed biannually and results of four measurements were analyzed. In the stereotactic coordinate system of GKRS, the radiation isocenter corresponds to a point of *x* = *y* = *z* = 100.0, where the *x*‐axis is defined from the right to the left of a patient, the *y*‐axis is defined from the posterior to the anterior, and the *z*‐axis is defined from the head to the feet. To evaluate the accuracy of patient couch movement, the same film test was repeated at an extreme position (40.0, 160.0, 100.0). The deviation of the radiation isocenter was also assessed daily using a diode detector installed on a vendor‐provided tool [Fig. 1(b)]. After measuring the dose distributions of the 4‐mm collimator along each axis, the radiation isocenter was automatically determined by the control system as the middle point of two full width at half maximum points, and deviations from 100.0 were recorded with 0.1‐mm precision.

### 2.B Accuracy of CBCT images

The CBCT of the GK Icon obtains images by rotating the C‐arm by 197° because the arm cannot pass through the patient couch [Fig. 1(a)]. The source‐to‐detector distance was 1000 mm, and the source‐to‐axis distance was 790 mm. The cone beam angle was 15°, and the reconstructed volume was 224 × 224 × 224 mm^3^. The CBCT image slice thickness was 0.5 mm, and the voxel size of an image was 0.5 × 0.5 × 0.5 mm^3^. The images obtained using the GK Icon CBCT have their own stereotactic coordinates, which are adjusted to coincide with the GKRS stereotactic coordinate system. This coordinate system can be assigned to other nonstereotactic tomography images through image coregistration procedures. The accuracy of the coordinate system was assessed daily using a vendor‐provided tool [Fig. 1(b)]. Four ball bearings with known stereotactic coordinates are located on the tool. After CBCT images of the tool were obtained, the control system automatically identified the locations of the four ball bearings and compared these locations with their predefined values. The one‐dimensional deviations of each ball bearing and overall maximum three‐dimensional deviation were recorded with 0.01‐mm precision.

Depending on the value of the weighted CT dose index (CTDI_w_), two presets were used for CBCT imaging: CTDI_w_ = 2.5 mGy and CTDI_w_ = 6.3 mGy. The CTDI_w_ values were measured and provided by the vendor. No additional parameters can be chosen by users for CBCT imaging. The operating peak voltage was 90 kVp. The tube current was 0.1 mA for CTDI_w_ = 2.5 mGy and 0.25 mA for CTDI_w_ = 6.3 mGy. The quality of the CBCT images was assessed following the protocols suggested by the vendor. The image resolution was assessed by measuring the number of line pairs per centimeter using a CatPhan^®^ 503 phantom [Phantom Laboratory, Salem, NY, USA, Fig. 1(c)]. The contrast to noise ratio (CNR) of the CBCT images was calculated using the mean and standard deviation of pixel values measured in a polystyrene insert and in a low‐density polyethylene (LDPE) insert located inside the phantom. The CNR was defined as noted in Eq. (1):

(1) $$CNR=\frac{I_{\mathrm{PS}}-I_{\mathrm{LDPD}}}{\sqrt{\sigma_{\mathrm{PS}}^{2}+\sigma_{\mathrm{PDPE}}^{2}}}$$

where $I_{\mathrm{PS}}$ is the mean pixel value measured in a square of interest of size 5 × 5 mm^2^ located in the polystyrene insert, $I_{\mathrm{LDPE}}$ is the mean pixel value measured in the LDPE insert, and $\sigma_{\mathrm{PS}}$ and $\sigma_{\mathrm{LDPE}}$ are standard deviations in each region for the polystyrene and LDPE, respectively. The uniformity of the CBCT images was calculated using Eq. (2):

(2) $$Uniformity\left(\%\right)=\frac{\left(I_{\mathrm{high}}+1000\right)-\left(I_{\mathrm{low}}+1000\right)}{I_{\mathrm{high}}+1000}\times 100$$

where $I_{\mathrm{high}}$ and $I_{\mathrm{low}}$ are the highest and lowest mean pixel values measured in five regions of interest 10 × 10 mm^2^ in size located at the center and at four points 45 mm from the center along the left, right, anterior, and posterior directions. The uniformity was measured for an image obtained at the homogeneous portion of the phantom. The image resolution, CNR, and uniformity were obtained twice a month.

### 2.C Accuracy of image coregistration

**Figure 2:**
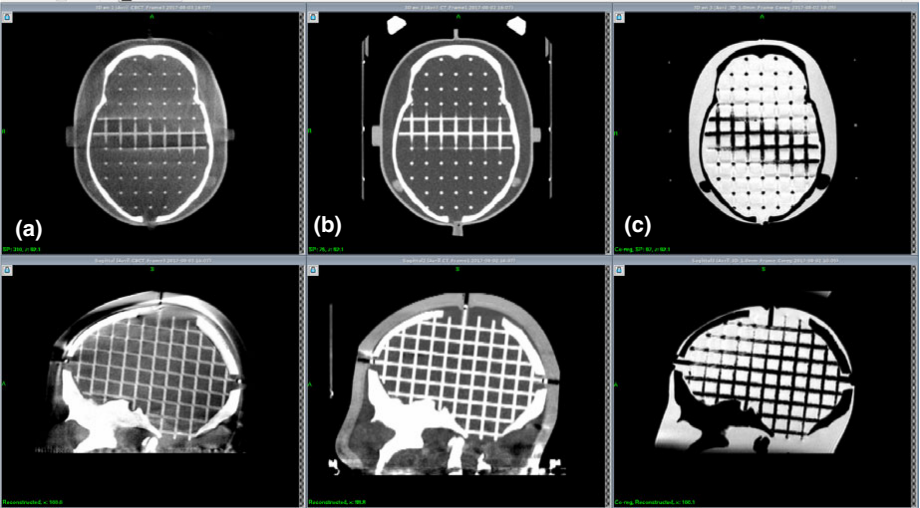
Examples of the CIRS 603a phantom images used for image coregistration error analysis. (a) GK Icon CBCT. (b) GE Discovery CT. (c) GE Signa 1.5 T MR.

The accuracy of stereotactic coordinates assigned by coregistration of the GK Icon CBCT images with clinical CT or MR images was evaluated using the commercial phantom CIRS 603a (CIRS Inc., Norfolk, VA, USA). Axial CT images of the phantom were obtained using a GE Discovery^TM^ CT750 HD (GE Medical Systems Inc., Waukesha, WI, USA) with a peak voltage of 120 kVp and a current of 250 mA in a helical mode. T1‐weighted axial 3D multiplanar gradient recall MR images were obtained using a GE Signa HDxT 1.5‐T MR instrument (GE Medical Systems Inc., Waukesha, WI, USA). The characteristics of the images are summarized in Table 1, and typical images are presented in Fig. 2. CBCT images were obtained without any frame on the phantom, but the CT and MR images were obtained after the phantom was fixed to a Leksell G‐frame (Elekta Instruments AB, Stockholm, Sweden) such that fiducial marker‐based coordinate systems could be assigned and compared with the coregistered systems. Coregistered coordinate systems were assigned to the CT and MR images by coregistering these images with the CBCT images using a gamma knife treatment planning system, Leksell Gamma Plan^®^ (LGP) version 11.0.1. Inside the phantom, 20 landmark points were arbitrarily chosen, and their positions were measured three times in each coordinate system. In the CBCT coordinate system, the ranges of the 20 landmarks were *x* = 54.1–144.2 mm, *y* = 10.4–161.8 mm, and *z* = 35.3–140.2 mm. One‐dimensional deviations along each axis were obtained by subtracting each coordinate from that of the CBCT system. A three‐dimensional deviation was defined as the root value of the squared sum of the three one‐dimensional deviations.

**Table 1:** Geometric characteristics of the images used for image coregistration analysis

	CBCT	CT	MR
Slice thickness (mm)	0.5	1.25	1.0
Resolution	448 × 448	512 × 512	512 × 512
Pixel size (mm^2^)	0.5 × 0.5	0.49 × 0.49	0.50 × 0.50

### 2.D Whole procedure test

**Figure 3:**
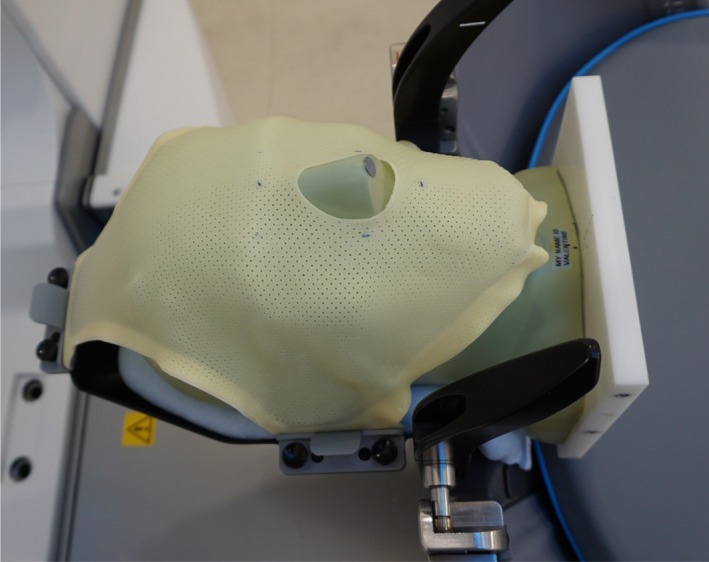
An anthropomorphic phantom is fixed to the GK Icon with a mask system for a whole procedure test of the accuracy of frameless GKRS.

To examine the overall geometrical accuracy of frameless GKRS, whole procedure tests were performed three times using EBT3 films and an anthropomorphic phantom, CIRS 605 (CIRS Inc., Norfolk, VA, USA). After a film was set in the phantom, clinical CT images were obtained. Then, the phantom was fixed with a mask as shown in Fig. 3, CBCT images were acquired with the CTDI_W_ = 6.3 mGy protocol, and the phantom was removed from the couch. The CT images were coregistered with the CBCT images, and the irradiation point was chosen as the center of the film, which was determined by fiducial markers on the film edges. The phantom was fixed again, and another series of CBCT images was obtained with the CTDI_W_ = 2.5 mGy protocol. Differences between phantom positions were automatically corrected after image coregistration, and irradiation was performed with a 4‐mm collimator. The film was scanned, and the radiological center was compared with the film center. Three films were used at each irradiation plane: the axial, coronal, and sagittal plane. In the first measurement, a GE LightSpeed Ultra CT (GE Medical Systems Inc., Waukesha, WI, USA) was used, and only the axial plane was examined.

### 2.E Accuracy of the HDMM system

**Figure 4:**
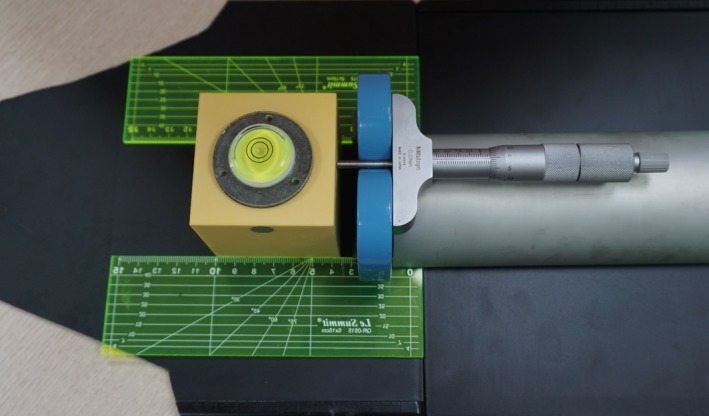
An in‐house device used to assess HDMM accuracy. The circular marker on the cube is the patient marker used to check patient movement. The cube was moved by a depth micrometer along a straight line guided by two plastic rulers.

In frameless GKRS, a marker is attached on the patient nose, and the motion is monitored by the HDMM system during irradiation. The HDMM system consists of an infrared stereoscopic camera, four reference markers, and one patient marker. The infrared camera is mounted onto an arm on the patient couch and continuously tracks the movement of the patient marker during treatment. The patient is immobilized with a thermoplastic mask, and the motion of the patient marker is tracked at a frequency of 20 Hz, with an accuracy of 0.15 mm.^16^ The accuracy of HDMM was verified using an in‐house device (Fig. 4). By moving a cube along the *x*‐ or *z*‐axis using a depth micrometer (Mitutoyo Corp., Kanakawa, Japan), the HDMM values were recorded. Five measurements were executed in each direction. The squared HDMM values were fitted with a second‐order polynomial of the micrometer movements.

### 2.F Statistical analysis

All statistical analyses were performed using the commercial package IBM^®^ SPSS^®^ Statistics version 22 (IBM Corp, Armonk, NY, USA). Pearson correlation analysis was used to investigate the relationship between two variables, and an independent *t* test or one‐way ANOVA was used to compare mean values. When the *P* value was less than or equal to 0.01, the difference was accepted as statistically meaningful. All of the mean values reported in this work are presented with one standard deviation.

## 3 RESULTS AND DISCUSSION

### 3.A Accuracy of the radiation isocenter and patient couch movement

**Figure 5:**
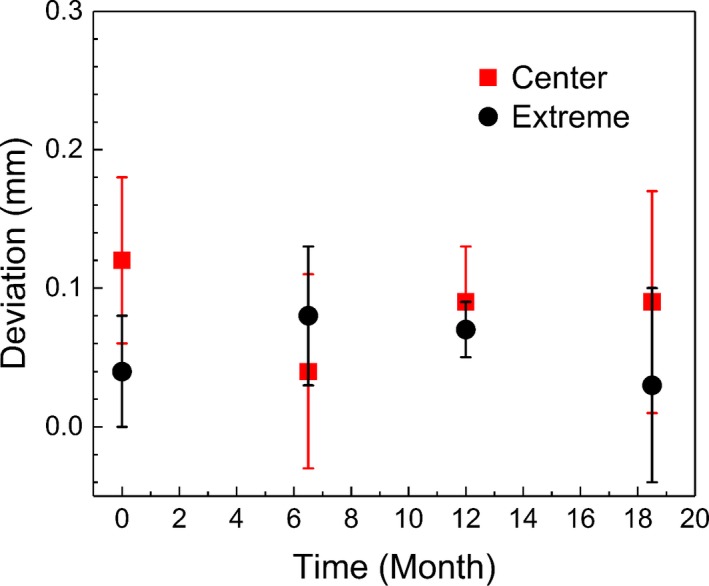
Deviations between the radiation iso‐center and the center of the patient positioning system. Radiochromic films were irradiated using a 4‐mm collimator at the center and at an extreme position (40, 160, 100).

Deviations between the radiation isocenter and the center of the PPS measured by films are presented in Fig. 5. The mean three‐dimensional deviation was 0.09 ± 0.03 mm and 0.06 ± 0.02 mm at the center and extreme point, respectively. The accuracy at the center point is consistent with the results of Novonty et al.^15^ and Zeverino et al.,^13^ who presented radial errors of 0.14 ± 0.06 mm and 0.13 ± 0.08 mm, respectively. The mean deviation of the radiation isocenter at the center evaluated with the diode detector was ‐0.1 ± 0.1 mm, 0.0 ± 0.0 mm, and 0.0 ± 0.0 mm along the *x*‐, *y*‐, and *z*‐axes, respectively. The mean three‐dimensional deviation was 0.1 ± 0.0 mm, which is consistent with the radial deviation of 0.1 ± 0.1 mm reported by Novotny et al.^15^ This deviation never exceeded 0.1 mm.

### 3.B Accuracy of CBCT images

**Figure 6:**
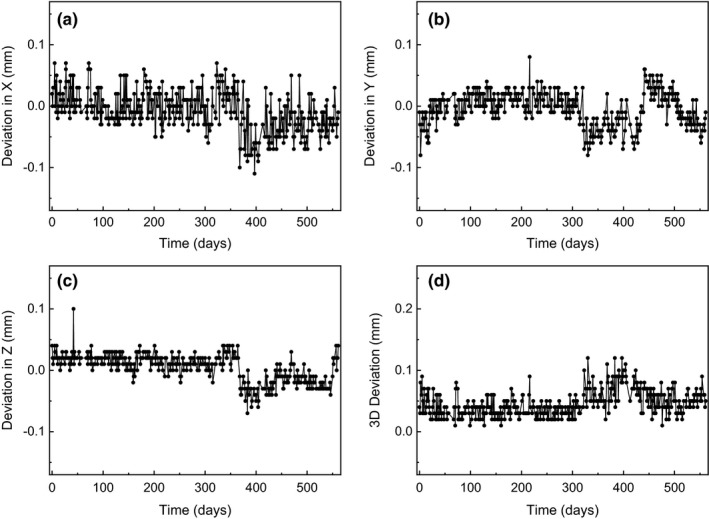
Variations of the mean one‐ and three‐dimensional deviations obtained for locations of the four ball bearings in the GK Icon CBCT.

Analysis of the one‐dimensional deviations of the four ball bearings showed that the one‐dimensional deviations along each axis were closely correlated (*p *<* *0.001), indicating that the CBCT images acted as a rigid body. Figure 6 presents daily variations of the mean one‐ and three‐dimensional deviations of the four bearings. The overall mean one‐dimensional deviation was −0.01 ± 0.03 mm, 0.00 ± 0.03 mm, and 0.00 ± 0.02 mm along the *x*‐, *y*‐, and *z*‐axes, respectively. The overall mean value of the three‐dimensional deviation was 0.04 ± 0.02 mm, and the mean value of the maximum deviation was 0.07 ± 0.02 mm. This value is less than the mean maximum deviation of 0.13 ± 0.05 mm reported by AlDahlawi et al.,^14^ which was measured during a 30‐day period after installation (*p *<* *0.001). Although the one‐dimensional deviations varied negatively over a period of days (*p *<* *0.006) and the three‐dimensional deviation increased (*p *<* *0.001), their correlations were not strong (|Correlationcoefficient| <0.615). The slope was less than 0.001 mm per day; thus, the three‐dimensional deviation did not exceed 0.12 mm. It is interesting to note the abrupt change in deviation at 1 year after installation when a PPS calibration was performed during an annual periodic management.

**Figure 7:**
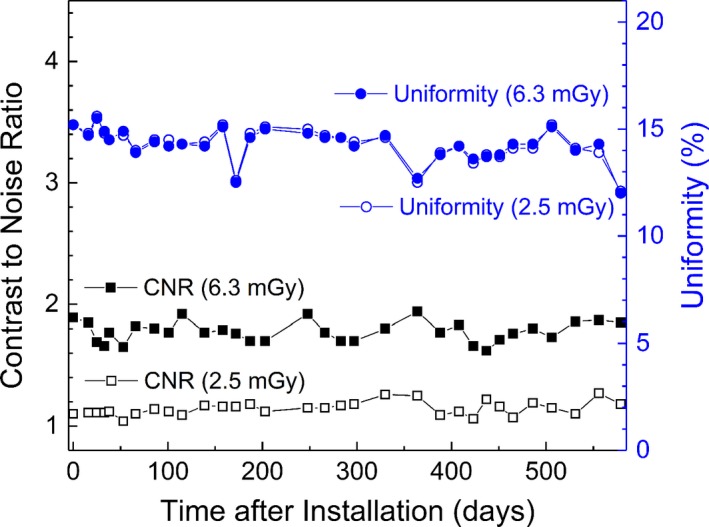
Variations of the contrast to noise ratio (CNR) and uniformity of the GK Icon CBCT images over time.

The image resolution assessed by the number of line pairs in the CBCT images of the CatPhan phantom remained constant throughout the investigation period (7 lp/cm for CTDI_w_ = 2.5 mGy and 8 lp/cm for CTDI_w_ = 6.3 mGy). One and a half years after installation, the mean CNR value was 1.14 ± 0.06 for CTDI_w_ = 2.5 mGy and 1.78 ± 0.08 for CTDI_w_ = 6.3 mGy. The mean uniformity was 14.3 ± 0.8% for both protocols. Variations of CNR and uniformity are presented in Fig. 7. The CNR values were better than those reported by Zevernio et al.^17^ (0.8 and 1.2, respectively, for each imaging preset). However, the uniformity of our system was worse than that of their system (9.2% and 8.8%, respectively, for each preset).^13^

### 3.C Accuracy of image coregistration

When stereotactic coordinate systems were defined using the fiducial markers, the mean fiducial marker registration error was 0.2 mm for the CT images and 0.4 mm for the MR images. The maximum registration error was 0.5 mm and 1.0 mm for the CT and MR images, respectively. The coordinate system of the CBCT images was used as the reference system in this work because its geometrical deviations were less than 0.12 mm, as described in the previous section. The coordinates of each point consistently exhibited a standard deviation of 0.1 mm. Image coregistration could be repeated with differences of the same order as the measurement error, that is, with a standard deviation of 0.1 mm. Table 2 presents the deviations measured for each image set for the fiducial marker‐based CT, fiducial marker‐based MR, coregistered CT, and coregistered MR images. No differences were noted between the fiducial marker‐based CT and coregistered CT coordinates (*p *=* *0.637). Both MR coordinates exhibited statistically larger deviations than the CT coordinates (*p *<* *0.001). When the MR images were coregistered with the CBCT images, their coordinates were more accurate than those assigned by the fiducial markers (*p *<* *0.001). This finding suggests that frameless GKRS with coregistered MR images may achieve better imaging accuracy than frame‐based GKRS. The errors in the fiducial marker‐based MR coordinates were primarily due to image distortion, marker location error, and marker registration algorithms.^18^ The MR image distortion, that is, the mean deviation of the fiducial marker‐based MR images compared with the fiducial marker‐based CT images, was 0.8 ± 0.3 mm, which is similar to the mean MR distortion error of 0.95 mm reported by Pappas et al.^19^

**Table 2:** The means and standard deviations of the fiducial marker‐based and coregistered stereotactic coordinate systems based on the CBCT system

	CT		MR	
	Fiducial	Coregistered	Fiducial	Coregistered
Deviation in X	0.1 ± 0.1	0.0 ± 0.1	0.1 ± 0.2	0.1 ± 0.2
Deviation in Y	0.0 ± 0.1	0.0 ± 0.1	0.7 ± 0.3	0.3 ± 0.3
Deviation in Z	0.0 ± 0.2	0.1 ± 0.2	0.3 ± 0.4	0.3 ± 0.3
3D deviation	0.2 ± 0.1	0.2 ± 0.1	0.9 ± 0.3	0.4 ± 0.2

### 3.D Whole procedure test

In the experiments performed to assess the end‐to‐end positional accuracy of frameless GKRS, the one‐dimensional deviations of the irradiated point from the planning point were 0.2 ± 0.2 mm, 0.3 ± 0.3 mm, and 0.3 ± 0.5 mm along the *x*‐, *y*‐, and *z*‐axes, respectively. The mean three‐dimensional deviation was 0.5 ± 0.6 mm. This result agrees with that of Ma et al.,^6^ who reported two‐dimensional deviations of 0.1–0.6 mm in a whole procedure test with CT images. The error observed in the whole procedure test was greater than the square root sum of the individual errors, the radiation isocenter positioning error, the CT image error, and the image coregistration error. A major source of the additional errors arose from locating the center of the film in the CT images. Given the finite image resolution and the ambiguity of the reconstructed coronal and sagittal images, the target could be defined with a three‐dimensional standard deviation of 0.2 mm. This result is similar to the whole procedure error of 0.48 ± 0.23 mm reported by Mack et al.^4^ Thus, the positional accuracy of frameless GKRS with CT images is similar to that of frame‐based GKRS with MR images. Although it is likely that frameless GKRS with MR images exhibited larger errors than frameless GKRS with CT images because their image coregistration error was increased, the whole procedure error is still expected to be at the submillimeter scale because minimal differences were noted between the MR and CT coregistration error (0.4 mm vs 0.2 mm).

### 3.E Accuracy of the HDMM system

**Figure 8:**
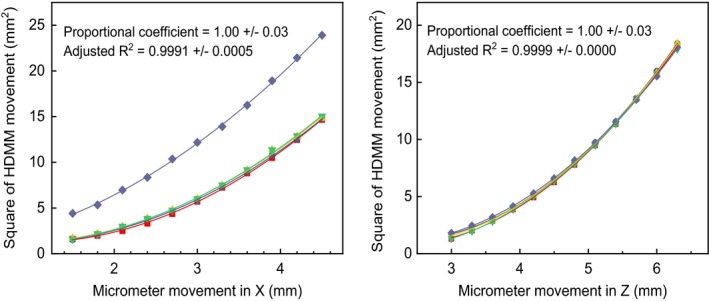
Relationship between the one‐dimensional motion of the micrometer and the movement measured by the HDMM system.

The proportional coefficient for the micrometer reading and the movement provided by the HDMM system was 1.00 ± 0.03 for both the *x*‐ and *z*‐axes, indicating that the HDMM system accurately measured the cube movement. Figure 8 presents the measurement data and their fitted lines. The isolated line in the *x*‐axis movement was attributed to the fact that its reference point was different from that of the other measurements. Of note, the accuracy of HDMM does not guarantee the accuracy of irradiation. The operator at irradiation sets the limit of allowed patient motion, and the actual movement range differs from patient to patient. Thus, the final accuracy of irradiation is different for each patient.

## 4 CONCLUSION

The positional accuracy of frameless GKRS was measured in the submillimeter range. The radiation isocenter accuracy, patient couch movement accuracy, and GK Icon CBCT accuracy were all approximately 0.1 mm and were stable for one and a half years. The coordinate system assigned to MR images through coregistration was more accurate than the system defined by fiducial markers. Possible patient motion during irradiation should be considered when evaluating the overall accuracy of frameless GKRS.

## CONFLICTS OF INTEREST

The authors have no relevant conflicts of interest to disclose.
